# Association between metabolic age and dementia risk: the role of genetic background

**DOI:** 10.1002/alz.088514

**Published:** 2025-01-09

**Authors:** Jiao Wang, Jie Guo, Huijie Huang, Abigail Dove, Yuyang Miao, Xiangyu Ma, Weili Xu

**Affiliations:** ^1^ Third Military Medical University, Chongqing, Chongqing China; ^2^ China Agricultural University, Beijing, Beijing China; ^3^ Aging Research Center, Karolinska Institutet, Stockholm Sweden; ^4^ School of Public Health, Tianjin Medical University, Tianjin China; ^5^ Tianjin Medical University General Hospital, Tianjin China

## Abstract

**Background:**

Growing evidence indicates that people with neurodegenerative diseases have altered metabolic status, but the association between metabolic age (MetAge), as assessed by circulating plasma metabolomics, and dementia remains unclear. We aimed to investigate the association between MetAge and risk of dementia and to explore whether genetic background plays a role in these associations.

**Method:**

From the UK Biobank, 153,436 dementia‐free adults aged ≥55 (mean age 62.08±4.07 years; 52.54% female) at baseline were followed up to 16 years to detect incident dementia. Dementia was diagnosed based on information from patient registry following international criteria. Nuclear magnetic resonance spectroscopy was used to measure 249 metabolites from plasma samples collected at baseline. MetAge (in years) was calculated based on these metabolites using the XGBoost machine learning algorithm (25.04 to 93.11 years) and tertiled (younger 25.0‐55.7 years, middle‐aged 55.7‐62.7 years, and older 62.7‐93.1 years). AD‐related polygenic risk score (PRS_AD_) was categorized as low, moderate, and high. Data were analyzed using Cox regression and Laplace regression.

**Result:**

During the follow‐up (median: 14.5, interquartile range: 13.6 – 15.3 years), 4,521 (3.0%) participants developed dementia. MetAge (as continuous variable) was dose‐dependently associated with dementia risk (hazard ratio [HR] and 95% confidence interval [CI] per 5‐year increase: 1.04 [1.02, 1.06]). Compared with younger MetAge, the HR (95% CI) for dementia was 1.13 [1.03, 1.23]. Older MetAge was also associated with earlier onset [10^th^ percentile difference [95% CI]: ‐0.89 [‐1.27, ‐0.50] years) of dementia. In joint‐effect analysis, the HR of dementia was 2.43 (95% CI 2.08, 2.83) among participants with older MetAge and moderate‐to‐high PRS_AD_, compared to those with younger MetAge and low PRS_AD_. There was an additive interaction between older MetAge and moderate‐to‐high PRS_AD_ on dementia (attributable proportion: 0.16 [0.01, 0.31]).

**Conclusion:**

Older MetAge is associated with a moderately increased risk of dementia and accelerates dementia onset by nearly 1 year, particularly among people with a high genetic susceptibility for dementia.

